# Molecular Targets Underlying the Anticancer Effects of Quercetin: An Update

**DOI:** 10.3390/nu8090529

**Published:** 2016-08-29

**Authors:** Fazlullah Khan, Kamal Niaz, Faheem Maqbool, Fatima Ismail Hassan, Mohammad Abdollahi, Kalyan C. Nagulapalli Venkata, Seyed Mohammad Nabavi, Anupam Bishayee

**Affiliations:** 1 Pharmaceutical Sciences Research Center, International Campus, Tehran University of Medical Sciences, Tehran 1417614411, Iran; fazlullahdr@gmail.com (F.K.); kamalniaz1989@gmail.com (K.N.); faheemthepharmacist@gmail.com (F.M.) pharm.fatee@yahoo.com (F.I.H.); 2 Department of Toxicology and Pharmacology, Faculty of Pharmacy, Tehran University of Medical Sciences, Tehran 1417614411, Iran; 3 Department of Pharmaceutical Sciences, College of Pharmacy, Larkin Health Sciences Institute, Miami, FL 33169, USA; kvenkata@Ularkin.org; 4 Applied Biotechnology Research Center, Baqiyatallah University of Medical Sciences, Tehran 1435916471, Iran; nabavi208@gmail.com

**Keywords:** quercetin, cancer prevention, diet, bioavailability, DNA damage, polyphenols

## Abstract

Quercetin, a medicinally important member of the flavonoid family, is one of the most prominent dietary antioxidants. It is present in a variety of foods—including fruits, vegetables, tea, wine, as well as other dietary supplements—and is responsible for various health benefits. Numerous pharmacological effects of quercetin include protection against diseases, such as osteoporosis, certain forms of malignant tumors, and pulmonary and cardiovascular disorders. Quercetin has the special ability of scavenging highly reactive species, such as hydrogen peroxide, superoxide anion, and hydroxyl radicals. These oxygen radicals are called reactive oxygen species, which can cause oxidative damage to cellular components, such as proteins, lipids, and deoxyribonucleic acid. Various oxygen radicals play important roles in pathophysiological and degenerative processes, such as aging. Subsequently, several studies have been performed to evaluate possible advantageous health effects of quercetin and to collect scientific evidence for these beneficial health claims. These studies also gather data in order to evaluate the exact mechanism(s) of action and toxicological effects of quercetin. The purpose of this review is to present and critically analyze molecular pathways underlying the anticancer effects of quercetin. Current limitations and future directions of research on this bioactive dietary polyphenol are also critically discussed.

## 1. Introduction

During the last decade, the proportion of scientific studies based on non-nutritive components of diet has increased. Such components are present in diet and have the ability to protect the body from the harmful effects of degenerative diseases, cancer, and cardiovascular ailments. Carotenoids and flavonoids, two distinct groups of phytochemicals, represent valuable constituents of food. Other dietary agents—such as phytoalexins, phenolic acids, indole-3-carbinol, and organosulfur compounds—are also important phytochemicals with interesting biological activities [1,2]. Phytochemicals are generally present in a variety of foods, fruits, vegetables, beverages, and many other food products and medicinally important herbal preparations. The important point that brings the attention of the scientists towards the naturally occurring compounds for the purpose of testing is the presence of numerous phytochemicals existing in plant-derived foods. There are a wide variety of biological activities which are still unknown for the majority of these compounds [3]. Plant-derived phytochemicals activate various cell signaling pathways that play key roles in the prevention of physiological disorders in the body, which are mainly responsible for the development of cancers, neurodegenerative and cardiovascular diseases [4,5]. Various scientific studies conducted on experimental animal models for the assessment of the exact mechanisms through which phytochemicals exert their actions provide a good and valuable description of how food supplements containing abundant amounts of phytochemicals exhibit protective roles against degenerative disorders [6]. It is noteworthy to find out that such plant-derived medicinally important constituents have the ability to demonstrate preventive and protective measures against pathological disorders.

Flavonoids are mostly present in nature in the form of benzo-γ-pyrone derivatives. These compounds are mostly present in a variety of plants, vegetables, and flowers. Flavonoids have diverse structural frameworks and play important roles in the body’s defense system. The beneficial effects of flavonoid-rich foods have been demonstrated by various studies [7]. Data collected from different clinical trials have tried to underscore the exact mode of action exerted by flavonoids. There is a need to evaluate new possible ways to understand the beneficial activities associated with the consumption of flavonoid-rich food in order to advance our knowledge about the possible beneficial action of plant extracts. There are 4000 types of different flavonoids found in nature with diverse subcategories, such as flavones, isoflavones, flavonones, and chalcones. Flavonoids possess important biological activities, such as anti-inflammatory, antioxidant, hepatoprotective, and antimicrobial properties [8].

Quercetin is a key member of the polyphenol family and is largely found in various vegetables and fruits, such as capers, lovage, dill, cilantro, onions, various berries (e.g., chokeberries, cranberries, and lingonberries), and apples. Quercetin is well known for its anticarcinogenic potential. The anticancer property of quercetin is due to various cell signaling mechanisms and its ability to inhibit enzymes responsible for the activation of carcinogens. Moreover, quercetin exerts anticancer effect by binding to cellular receptors and proteins [9,10].

Several previous publications [11,12,13,14,15,16] present an excellent overview of research related to the therapeutic application of quercetin in cancer prevention and treatment. Nevertheless, there exists a need for a systematic, up-to-date, and critical evaluation of literature to understand biochemical and molecular mechanisms of the anticancer action of quercetin. In this review article, first we focused on chemical reactivity of quercetin and related analogs. Secondly, we discussed the molecular targets as well as signaling pathways implicated in anticancer and cancer preventive potential of quercetin. Thirdly, we presented epidemiological evidences regarding quercetin consumption and cancer occurrence. Finally, we discussed future directions of research to understand the full potential of quercetin in cancer prevention and treatment.

## 2. Bibliographic Search

**Figure 1:**
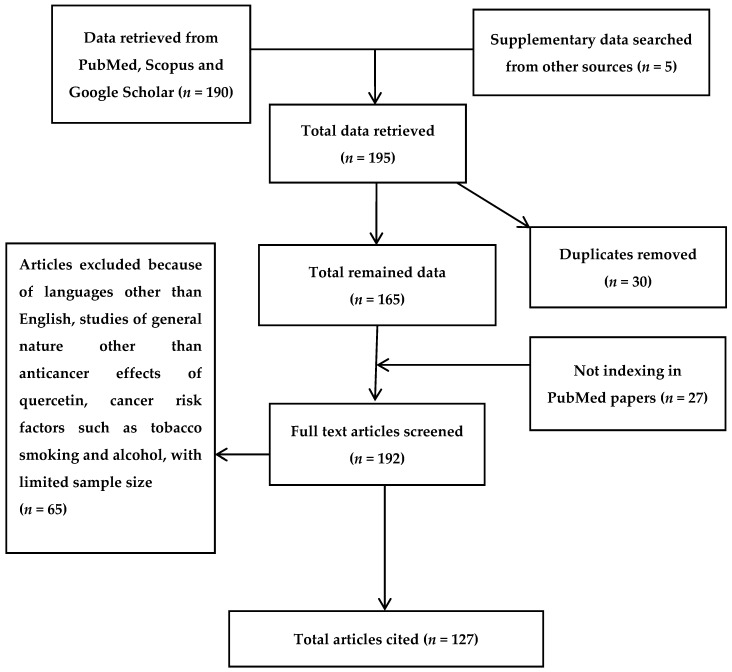
Flow diagram of included studies. The number of citations and resource materials that have been screened, excluded and/or included in this review is indicated in parenthesis.

The scientific information gathered in this review was collected by widespread search of several electronic databases, including PubMed, Scopus, Medline, Web of Science, EBASE, and Google Scholar. The criteria for the exclusion of articles was the language of reports being other than English, reports with unavailable abstracts, studies related to quercetin effects apart from its anticancer profile, and studies which showed the linkage between cancer and cancer risk factors, such as tobacco smoking and alcohol consumption. Various appropriate articles not indexed by PubMed were also considered, and 27 such reports which fulfilled the criteria for inclusion were further recovered from Google Scholar. Therefore, the total number of included articles in this review is 127 (Figure 1).

## 3. Chemistry of Quercetin and Its Analogs

**Figure 2:**
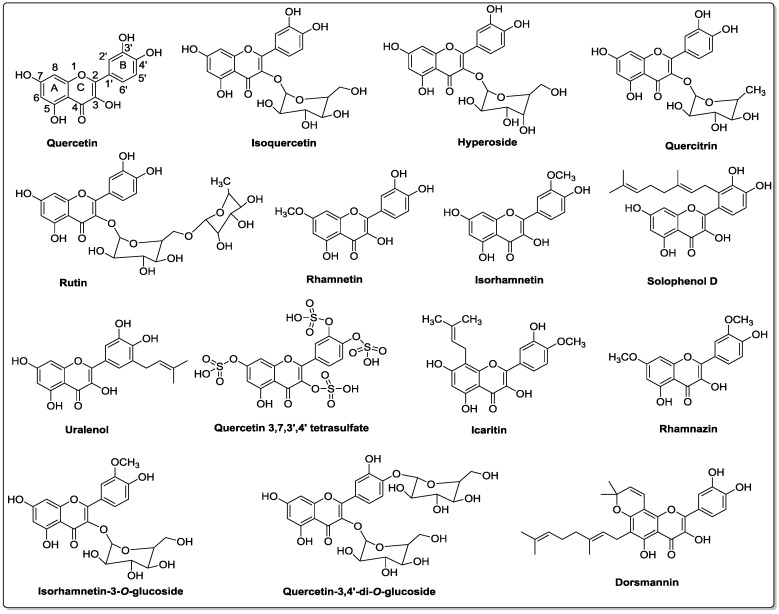
Structures of quercetin and its derivatives [19,20,21,22,23,24].

Quercetin is a polyphenolic secondary metabolite that belongs to the flavonol class of flavonoids. It is characterized by a benzo-(γ)-pyrone skeletal structure with C6-C3-C6 carbon framework, consisting of two benzene rings, A and B, linked by a three-carbon pyrone ring C as shown in Figure 2 [17]. Quercetin is referred to as pentahydroxyflavonol due to the presence of five hydroxyl groups on its flavonol skeletal framework at 3, 3′, 4′, 5, and 7 carbons [12]. The wide range of biochemical and pharmacological activities of quercetin and its metabolites is due to the relative substitution of various functional groups on the flavonol molecule [18].

Phytochemical investigations of various plant extracts have revealed that quercetin can exist in a free state as an aglycone, or as its derivative by conjugating with: (1) carbohydrates as quercetin glycosides, (2) lipids as prenylated quercetin, (3) alcohols as quercetin ethers, and (4) a sulfate group as quercetin sulfate [19].

Quercetin glycosides are formed through the *O*-glycosidic bond between a sugar and the hydroxyl group of quercetin molecule, and the general glycosylation site on the quercetin molecule is at the 3-hydroxyl position. However, glycosylation of other hydroxyl groups has also been reported [20]. The sugar moieties can be monosaccharides, disaccharides, or polysaccharides, and the most common carbohydrate substituents are glucose, galactose, rhamnose, and xylose. Isoquercetin, hyperoside, quercitrin, and rutin are a few of the most abundant and well-studied quercetin glycosides (Figure 2) [21].

Quercetin methyl ethers are one of the most widely studied natural pigments, and they are formed through the conjugation of the quercetin hydroxyl group with alcohol, generally methanol. Quercetin ethers can exist in various configurations, from mono-ethers to penta-ethers, with substitution on all the hydroxyl groups of quercetin molecule. Rhamnetin, isorhamnetin, and rhamnazin are a few representatives of quercetin ether analogs [19].

Prenylflavonols are an important group of molecules belonging to the flavonol subclass. They are well-known for their wide range of biological activities, such as antioxidant, antibacterial, anti-inflammatory, and anticancer properties [22]. Structurally, in *C*-prenylflavonols, prenyl groups are attached to the carbon atom of the flavonol skeleton. In the past few years, prenylated quercetin analogs, such as solophenol D and uralenol, have gained a lot of attention due to their antibacterial properties. It has been reported that prenylation may enhance the biological functions of quercetin by increasing its hydrophobicity and bioavailability [23,24].

In addition to the sugar, lipid, and alcohol derivatives, quercetin also exists as sulfate conjugate in nature. Quercetin 3,7,3′,4′-tetrasulfate, isolated from the leaves *Flaveria bidentis,* has shown remarkable anticoagulant properties [25]. The multisubstituted derivatives, such as icaritin, isorhamnetin 3-*O-*glucoside, quercetin-3,4′-di-*O-*glucoside, and dorsmannin, can form from the combination of same or different functional groups. The number and nature of these functional group substitutions have profound effects on the physicochemical properties and biological effects of quercetin analogs [26].

The best described biochemical property of quercetin is its ability to act as an antioxidant. The antioxidant activity and free radical scavenging properties of quercetin are attributed to its chemical structure [27]. There are three important structural features: (1) catechol functionality (ortho-dihydroxyl) on B ring, (2) a Δ^2^ double bond adjacent to a 4-oxo group in pyrone C ring, and (3) hydroxyl groups at C-3 and C-5 carbons in the benzopyrone AC ring [28]. The structural variables—such as configuration, substitution, and number of hydroxyl groups—greatly influence the mechanisms involved in antioxidant activity, like their ability to scavenge radical species and their ability to chelate metals [29].

Quercetin also inhibits the lipid peroxidation process, a common consequence of oxidative stress, and consequently protects against lipid membrane damage [30]. Due to its lower redox potential, quercetin is able to reduce highly oxidizing free radicals, such as superoxide and peroxide radicals. Because of its ability to chelate metal ions, quercetin can inhibit the generation of free radicals [28].

## 4. Bioavailability and Metabolism of Quercetin

In order to estimate the efficacy of quercetin in terms of its anticarcinogenic effect, it is important to understand the bioavailability of quercetin as well as its intestinal absorption and metabolism conversion rate. When quercetin was administered intravenously to rodents, it immediately disappeared from the plasma. It was evident from this experiment that quercetin was rapidly metabolized and eliminated from the body through urine and no evidence was observed regarding the storage of quercetin inside the tissues and body fluids. Previously, there was a common belief about the excretion of quercetin into feces without being absorbed by the intestine, but it is evident from recent studies that an excessive amount of quercetin found in foods is likely to be absorbed from the intestine and subsequently converted to its respective metabolites [31]. In the transportation of the metabolites of quercetin, the body’s lymphatic system is also involved [32]. Repeated intake of onion resulted in accumulation of quercetin metabolites in various tissues and blood, which reached a total plasma concentration of 0.6 µM after 1 week. Hence, it is important to keep the plasma quercetin metabolite concentration at an acceptable and significant level [33].

It is evident from studies conducted recently that the metabolites of quercetin were rapidly distributed among various organs at low levels after intake of dietary quercetin for a long time [34]. It is also evident that regular consumption of dietary quercetin results in the storage of metabolites throughout the body [35]. Generally, the conversion of quercetin to its metabolic derivatives decreases its free radical scavenging activity, but there are some metabolic derivatives of quercetin, which are capable of removing the reactive species from the body. Moreover, during the process of inflammation, quercetin-3-glucuronide is metabolized, resulting in the accumulation of quercetin aglycone [36]. Recent studies showed that glucuronide, a more active form of aglycone metabolite of quercetin, was used for the incorporation into macrophages [34]. This study showed those actions of quercetin metabolites which are mostly site-specific in nature and are recommended for inflammatory conditions. The modified forms of quercetin are present in human blood and stored in inactive forms, which are further converted into active residues and ultimately converted into the active constituents to exert their actions at specific target sites.

## 5. Protective Effects of Quercetin

Quercetin and its metabolites are crucial due to their physiological functions as well as their role in the elimination of cancerous cells. Therefore, it is important to further investigate this specialized aspect of quercetin in terms of protection against cancer and other degenerative disorders, as these ailments are the leading causes of death throughout the world.

It was investigated recently that by influencing the pentose phosphate pathway with the production of CYP450, the preventive environment for cytochrome c-mediated apoptosis can be maintained. This cytochrome is an important part of the electron transport chain and is released from mitochondria during the apoptosis process. Therefore, the cancerous cells keep constant control over cell death through the release of cytochrome c [37]. These metabolic changes are important for the survival of cells for a longer time and also for the spreading of cancer cells [38]. Various studies have been performed to evaluate the pharmacological actions of quercetin in biological systems in order to investigate the precise mode of action of quercetin [39,40]. Therefore, it is evident from these studies that quercetin and its metabolites, which are present in various plants, play crucial roles in the protection against cancer and oxidative stress. When PC-12 cells were treated with nerve growth factor (NGF), the cells ceased to multiply and began to extend branching varicose processes similar to those produced by sympathetic neurons in primary cell culture [41]. Quercetin exhibited NGF-like action when it came in contact with PC-12 cells. Quercetin also causes cell differentiation similar to that caused by NGF [42]. Although the exact mechanism is still unclear, the well-characterized NGF-inducing capacity is more likely related to the differentiation-inducing effects of quercetin [43]. The protective effects of quercetin have also been observed in primary cultures [39]. Quercetin increased the rate of survival of cells when it was administered 24 h before the oxidative stress. In a cell culture model, it was reported that quercetin internalization into neurons happened rapidly, and a neuroprotective pathway involved Nrf2-dependent variation of the GSH redox system was observed [44].

When the aqueous quercetin was administered in experimental animals, there was a significant reduction in brain ischemic lesions [45]. Quercetin has been used in a variety of studies to evaluate its protective effects. In one study, the anticancer effects of quercetin were studied in animal models. The investigators examined the physiological changes after they administered colchicine by intracerebroventricular route [46].

In a similar study, quercetin was administered to mice for a period of seven days through intraperitoneal (i.p.) route. After administration of quercetin, memory improvement was observed in mice [47]. In this study the developmental changes were linked with the inhibition of cyclooxygenase 2 enzyme. It was observed that there was a noteworthy improvement in the learning ability of mice in comparison with the control group of mice [48]. Quercetin also increased the activity of superoxide dismutase (SOD) and lowered the level of malondialdehyde. Quercetin and other flavonoids acted as prodrugs and were metabolized into active hydroxyphenyl acetic acid metabolites by microflora in the intestine [49]. The protective effects of quercetin were observed in rats with known evidence of cerebral ischemia [50]. By administering two consecutive doses of quercetin, the memory problem in rats—which was induced by repeated cerebral ischemia—was improved and the level of cell death was reduced in the region I of hippocampus proper area (CA1). In another study, when quercetin was administered through i.p. route to rats with spinal cord injuries for a period of 10 days, half of the rats started walking [51].

In a study evaluating the penetration of quercetin across the blood–brain barrier, it was observed that quercetin induced various changes within different brain regions based on an in situ brain perfusion model in rats [52].

## 6. Molecular Mechanisms of Quercetin

Quercetin is used for therapeutic purposes in various disorders due to its antioxidant capability The reactive oxygen species (ROS) scavenging activity is attributed to a change in OH^−^ ions, which has a relation to electron exchange [53]. Catechol oxidative agents, such as semiquinones and quinones formed due to quercetin, alter redox homeostasis and inhibit primary positive effects [27]. In vitro study predicted that due to this special feature quercetin has a protective role in the nervous system. In the current scenario, the neuroprotective activity of quercetin cannot be ignored and additional in vivo studies should be investigated with different humanized animal models to translate the efficacy. Quercetin exerts its antioxidant activity by competitively inhibiting the xanthine oxidase enzyme and noncompetitively blocking the xanthine dehydrogenase enzyme. The inhibitory capabilities are due to its flavonoid structure rather than to its antioxidant potential [54].

### 6.1. HMGB1 Signaling Pathway

High-mobility group box protein 1 (HMGB1) is a nuclear protein which is highly preserved. It is secreted by the action of macrophages previously activated and it works as a crucial “late” facilitator of fatal endotoxemia and sepsis formation [55]. The HMGB1 protein, which is present outside the cell, can motivate the release of tumor necrosis factor-α (TNF-α), interleukin-1β (IL-1β), and other inflammatory mediators from monocytes [55,56]. Quercetin stimulates the inhibition of HMGB1-induced TNF-α and IL-1β mRNA expression, which suggests that quercetin modulates cell signaling that in turn regulates the action of proinflammatory cytokines. The activation of mitogen-activated protein kinase (MAPK) signaling pathway is a significant step in the HMGB1-induced gene expression process, which causes the release of inflammatory cytokines—such as TNF-α, and IL-1β—inside macrophages, neutrophils, and endothelial cells. HMGB1-induced cytokine release partially interferes with MAPK pathways. HMGB1 or lipopolysaccharides (LPS) time-dependently induce phosphorylation of p38, c-Jun *N*–terminal kinase, and extracellular signal-regulated kinase in macrophages. Quercetin considerably inhibits HMGB1- or LPS-induced phosphorylation of each kinase [57]. Apart from MAPK activation, the nuclear factor-κB (NF-κB) signal transduction pathway is also involved in HMGB1-induced cellular activation, and NF-κB-dependent transcriptional activity is very important for cytokine expression [58,59]. In cells, NF-κB subunits (p50 and p65) exist as inactive trimers in the cytosol through the interaction with IκBα, which is the most important member of the IκB family [60]. Quercetin significantly inhibits IκBα degradation and nuclear translocation of NF-κB p65. Therefore, after stimulation with HMGB1 or LPS, p65, the key activator of NF-κB-regulated transcription, becomes available to NF-κB-regulated genes in the nucleus and nuclear localization is most effectively inhibited by quercetin.

### 6.2. Thymic Stromal Lymphopoietin (TSLP) Activation

The level of TSLP, which is an epithelial-derived cytokine with a role in T helper (Th) cells’ Type-2 immunity, is considerably increased in human skin as well as blood. The TSLP signaling process is initiated through proteins, including 1L-7 chain of receptor, which has potential to enhance the B lymphocyte activation and dendritic cells [61]. Primary skin keratinocytes are mostly responsible for expressing the TSLP in smooth muscle and lung connective tissues. TSLP contributes its main biological role by influencing various cells [62]. It is evident that TSLP has the potential to activate both CD4^+^ T and CD8^+^ cells along with other B lymphocytes’ differentiation, which in turn promotes the release and activation of chemokines. Additionally, it can enhance the secretory mechanism of the Th2 cytokines from mast cells. During the binding of TSLP with respective receptors, various signaling pathways are activated [63]. It has been reported in a recent study that due to the activation of these receptors, there is a marked promotion in the phosphorylation process of Janus kinase-signal transducers and activators of transcription (JAK-STAT) signaling, which further causes skin inflammation [64]. Thus, targeting the above signaling pathway is a viable approach to design a treatment plan for various inflammatory diseases, including cancer.

### 6.3. JAK-STAT Signaling Pathway

JAK-STAT signaling pathway is a typical signal transduction pathway for various types of inflammatory cytokines and growth regulatory factors. The binding of ligands to their respective receptors leads to the activation of JAK, which further increases the phosphorylation process and hence leads to the activation of STAT. The STATs, which are already activated, enter the nucleus, where they start the regulation of gene expression [65]. Studies have shown that the activated mast cells stimulate the formation of the Th2 cytokines and decrease the secretory mechanism of Th1 cytokines. JAK-STAT signaling is activated by mast cells, which in turn activate the IL-13 production in the Th2 cell line [66].

**Figure 3:**
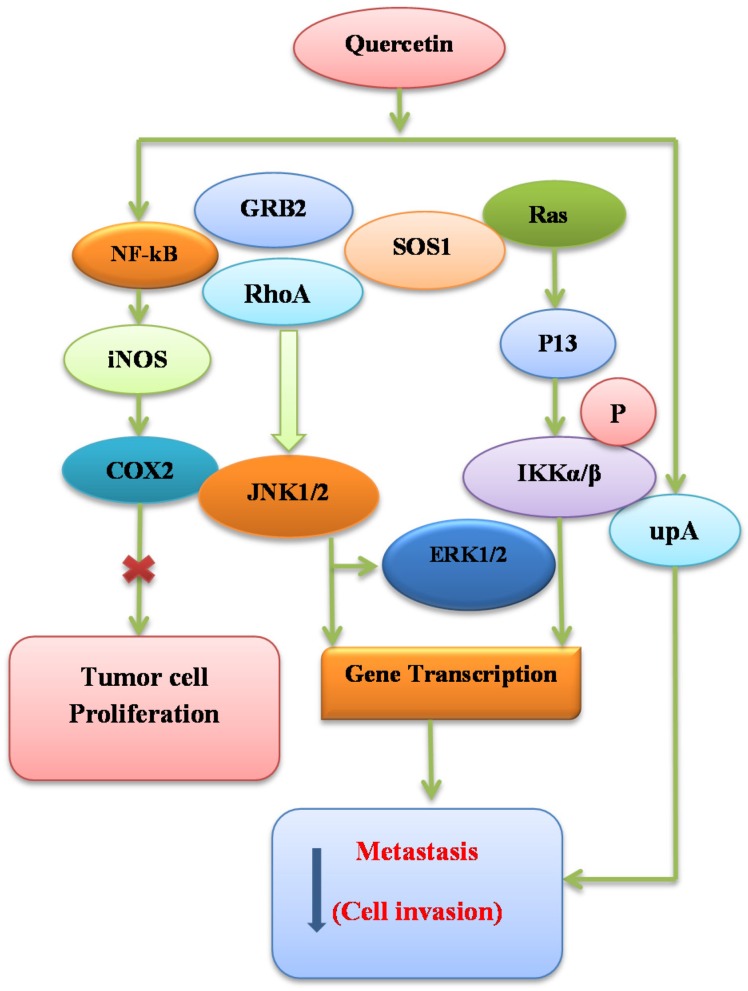
Anticancer pathways and mechanisms of quercetin.

Quercetin has the ability to actively inhibit the JAK-STAT signaling pathway in various inflammatory disorders. Furthermore, treatment of activated T cells with quercetin in vitro inhibited the interleukin-12 (IL-12)-induced phosphorylation of JAK2, tyrosine kinase-2 (TYK2), STAT3, and STAT4, which result in decreased levels of T cell propagation and Th1 variation [67]. Therefore, these anti-inflammatory and antiapoptotic properties of quercetin have a key role in the reduction of cancer by controlling the toll-like receptor-2 (TLR2) and JAK2/STAT3 pathway and causing the inhibition of STAT3 tyrosine phosphorylation within inflammatory cells [68]. Pretreatment of cholangiocarcinoma cells with quercetin inhibited the cytokine-mediated upregulation of inducible nitric oxide synthase (iNOS) and expression of intercellular adhesion molecule-1 (ICAM-1) in the JAK/STAT cascade pathway. Also, quercetin blocked the activation of inflammatory cytokine interleukin-6 and interferon-γ [69]. It was reported that LPS-induced STAT1 activation was inhibited by quercetin in combination with its profound inhibitory effects on iNOS and NF-κB expression, which are persistently involved in activation of interleukin-2 (IL-2) as shown in Figure 3 [70].

## 7. Anticancer Effects of Quercetin

Vegetables and fruits are rich sources of phytochemicals with significant potential to prevent cancer due to the presence of abundant antioxidants. Among polyphenols, quercetin is the most important and naturally occurring cancer-preventing agent. The importance of dietary quercetin is due to its antioxidant potential and anti-inflammatory effects [47]. The cancer preventive and therapeutic effects of quercetin have been demonstrated through in vitro (Table 1) as well as in vivo (Table 2) experimental findings.

Quercetin, when used in pharmacologically safe doses, inhibits the phosphatidylinositol 3-kinase (PI3K)-Akt/PKB (protein kinase B) pathway in cancer cells [81]. Both Raf and MAPK/extracellular signal-regulated kinase (ERK) kinase (MEK) act as direct targets for quercetin, leading to the potential to decrease MEK1 activity more powerfully when compared to PD098059, which is a specific MEK inhibitor. Quercetin donates a hydrogen bond to the main amide group of Ser-212, which is known to stabilize the inactive conformation of the activated loop of MEK1 [82]. When a dose of 10 g quercetin/kg was administered to rats for 11 weeks, physiological changes were observed inside the rats’ colons. This study indicated that quercetin extensively downregulated the potential oncogenic MAPK signaling in vivo [83]. Various in vitro studies have demonstrated that quercetin plays a key role in cancer prevention and tumor suppression in different cell lines [84]. The doses of quercetin that showed anticancer effects in vitro were ranging from 3 to 50 µM [85]. The cancer prevention properties of quercetin in vivo studies have been confirmed in colon cancer [11]. Furthermore, quercetin has been shown to exhibit anticancer effects in melanoma [86]. The inhibition of tumor growth by quercetin was evaluated when it was administered as a food supplement to experimental models. However, contradictory results have been reported in the literature [87].

### 7.1. Binding Ability of Quercetin to SEK1–JNK1/2 and MEK1–ERK1/2

Studies have shown that various pathways are the possible molecular targets of quercetin to inhibit inflammatory responses as shown in Figure 3. Quercetin chemically binds to protein kinase as evidenced by the bead-bound pull-down assay, which has been recognized as a potent screening tool. Quercetin binds with SAPK/ERK kinase 1 (SEK1), c-Jun *N*-terminal kinase 1/2 (JNK1/2), MEK1 and ERK1/2 [93].

Several studies have provided useful evidences that there are many hydrogen bonds between different hydroxyl groups of quercetin and amino acid residues of SEK1–JNK1/2 and MEK1–ERK1/2. The ERK1/2 is part of a MAPK cascade, which consists of consecutively functioning kinases, such as Raf, MEK, and ERK1/2 [94]. The active ERK1/2 induces reprogramming events related to gene expression by actively phosphorylating diverse intracellular molecular target proteins and other transcription factors, and hence potentiates cellular growth, spreading, and antiapoptotic properties.

### 7.2. Cellular Senescence Induction and Telomerase Inhibition

It is a process of irreversible cell aging that occurs in most of the normal cells in response to the restriction of telomerase enzymes or due to changes in the three-dimensional structure of telomerase. The cellular death activity is also associated with oncogenic activation or stress caused by oxidation [95]. The process of senescence induction by phytochemicals is a new alternative approach to chemoprevention. In a recent study, it was confirmed that both quercetin and resveratrol induced the cell death process even in very low doses in cells which showed resistance to glioma formation [96]. Despite the fact that there was no proper identification of a molecular target, there was a marked decrease in Akt phosphorylation. In the senescence induction process, quercetin also targeted the telomerase induction in eluding the replicate immortality.

Telomerases are specialized DNA polymerases having the ability to join the repeating parts of the telomerase enzymes with the ends of the DNA strands. The enzyme telomerase is significantly expressed at certain intervals in the majority of the cells, including the excessively proliferating human cells. The presence of telomerase activity is closely related to cell death resistance [95]. Quercetin and other polyphenols, including epigallocatechin-3-gallate (EGCG), inhibit the activity of telomerase in a cell-free system. The telomerase inhibitory effect was confirmed using adenocarcinoma and breast carcinoma patients [97].

### 7.3. Cell Death Induction Activity 

Programmed cell death is an important mechanism which is activated to eliminate cancer cells in the body [95]. There are extrinsic and intrinsic pathways which control the cell death activity in the body, and these pathways are under the influence of cytokines, which act by binding with tumor necrosis factor receptors (TNF-R). The cytokines are large molecules which are mostly involved in the development of immunity [98].

Quercetin bypasses the cell damage resistance through various mechanisms. One example of quercetin action is evident in lymphocytic leukemia cell line. Quercetin is introduced to the body with minimum toxic concentrations to induce the apoptotic process. From this study, it is evident that quercetin induces the apoptotic process when combined with antibodies for the enhancement of immunity [99].

At molecular levels, quercetin acts by lowering the ROS inside the cell. This property of scavenging free radical species is a unique function of quercetin among flavonoid derivatives [100]. Apoptosis-inducing activity of quercetin has been confirmed in leukemia and also in cells which are resistant to TNF-related apoptosis-inducing ligand (TRAIL)-induced apoptosis [101].

### 7.4. Interactions of Quercetin with Cellular Receptors

Quercetin binds with different receptors present throughout the body and shows its anticancer properties. In a recent study, it was concluded that both Raf and MEK are molecular targets of quercetin in the prevention and treatment of cancer [102]. Whether quercetin has direct interactions with cell receptors is still unclear, but there are reports that aryl hydrocarbon receptor (AhR) is a known molecular target receptor for the majority of flavonoids, including quercetin. The AhR receptor is a ligand-gated transcription factor which is activated by the interaction with synthetic and natural chemicals [45,103]. Similarly, AhR is responsible for the regulation and expression of cytochrome P-450 (CYP) 1 family, and this family is fully capable of activating procarcinogens. In the biotransformation of polycyclic aromatic hydrocarbons (PAHs) to metabolites, which are carcinogenic in nature, the CYP1 family members are actively involved [104]. The process of biotransformation is closely associated with cancer development in the lung. Also, a high level of CYP1 is responsible for colon cancer, a major cause of cancer-related death [105].

Quercetin inhibits the transformation of AhR and protects the cells from the toxicity induced by dioxins [106]. The IC_50_ values of quercetin 3-*O*-β-d-glucuronide and quercetin 4′-*O*-β-d-glucuronide were 42.6 and 181 µM, respectively. It is indicated from this result that aglycones have stronger antagonistic activity than glucuronides and other metabolites. Quercetin also blocks the biotransformation of AhR in rat hepatocytes. It is noteworthy that the inhibitory effect of quercetin is much stronger than the effects of α-naphthoflavone, a well-known antagonist for AhR [107]. It is evident from these results that quercetin is a strong antagonist for AhR and hence exerts its pharmacological effects against carcinogenicity developed by PAHs. Quercetin also interacts with other receptors, which are involved in the prevention of cancer, but the exact mechanism is still unclear [108]. Quercetin has no role in the increase or decrease and distribution of estrogen receptors-beta isoforms in breast cancer [109]. Quercetin has been shown to possess inhibitory effects in human prostate cancer, and this effect is linked to the expression of androgen receptors [110]. It has been suggested in a recent report that a transcription factor is involved in quercetin-mediated inhibition of androgenic receptor [111].

### 7.5. Signal Transduction Modification

**Figure 4:**
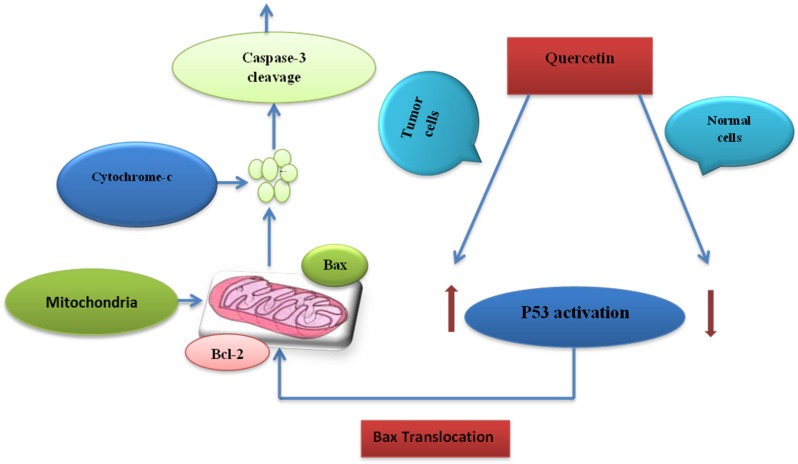
Modulation of mitochondrial apoptotic signaling pathways by quercetin. Quercetin induces p53 activation resulting in upregulation of Bax and downregulation of Bcl-2 in tumor cells. This leads to caspase activation and ultimately apoptotic cell death.

There are various reports which document modulatory effects of quercetin and other flavonoids on signal transduction pathways. The use of quercetin enhances the cell death process in HepG2 human hepatoma cells. There are two mechanisms involved in this process; one is the activation of caspase-3 and caspase-9, but not caspase-8. The other mechanism involves an increase in the translocation of proapoptotic Bax to the membrane of mitochondria [112]. In a similar study, it has been shown that quercetin causes the cleavage of polymerase and also potentiates the upregulation of Bax (Figure 4). Quercetin decreases the levels of key oncogenic protein Ras in cancer cells and blocks the cell proliferation and survival [113].

Quercetin has been shown to cause changes in apoptosis in mesangial cells by inhibiting the activation of JNK and other ERKs pathways. It is also clear that there is no significant effect on the level of p38 MAPK [114]. In a similar study, it is reported that quercetin caused the inhibition of phosphorylation of p38 and Bcl2. This property of quercetin is useful as it may stop the apoptotic process [115]. The action of quercetin may be considered as short-term or long-term. The short-term effect causes scavenging of free radicals and it is mostly antioxidative and antiapoptotic in nature, while the long term effect is pro-oxidative [116]. The proapoptotic action of quercetin is linked mostly to a decreased level of glutamate-stimulating hormones (GSH). It is evident that GSH plays an important role in determining the antioxidant nature of flavonoids. The action of quercetin may depend upon the concentrations of quercetin in cell culture medium [117].

How to determine the accurate mechanism through which quercetin exerts its effect in controlling the signal transduction pathway is still unclear. There are few studies on the mechanisms of action of quercetin, and the specific targets of quercetin are not well known [118]. Apart from the findings mentioned above, it has been reported that the MEK/ERK pathway is activated by quercetin during the process of programmed cell death in human lung cancer. This contradiction may be because of the basic difference in investigational conditions, including the use of various cell lines [119]. Quercetin is important in the regulation of signal transduction pathways, and such pathways are crucial in the production of inflammatory mediators. Quercetin also decreases the LPS formation of cytokinase, and this enzyme causes the inhibition of iNOS by further suppressing ERK and p38 MAPK [120]. Moreover, quercetin significantly reduces the half-life of Ras protein, which is oncogenic in nature, but no significant action was reported when the cells were treated with proteasome inhibitor [113].

## 8. Epidemiological Studies about Quercetin

Emerging studies suggest that intake of fruits and vegetables decrease the risk of human carcinomas, including colon, breast, bladder, stomach, and lung cancer [121]. A plethora of phytochemicals present in plant-based food products are consumed by humans on a daily basis. However, it is still unknown which one among these phytoconstituents is responsible for protective action against cancer. The most studied phytochemicals for anticancer potential are flavonoids [122]. A few studies indicate an opposite relation among the dietary consumption of polyphenols and cancer risk [123]. However, Hertog and his coworkers [124] assert that there is no association between the intake of flavonol or flavone derivatives and cancer-related mortality rates.

Regarding quercetin, an opposite co-relation exists between the consumption of quercetin found in foods and the development of lung cancer caused by smoking [125]. A recent multiethnic clinical study has provided evidence about the cancer preventive effects of quercetin in the progression of pancreatic cancer in individuals who were chain smokers and nonsmokers. However, the effect was more pronounced in smokers compared to nonsmokers, as quercetin imparted its antioxidant activity in smokers who had increased oxidative stress relative to nonsmokers [126]. It is evident from many studies that quercetin has beneficial effects against cancer risks. In a study, Gates and coworkers [127] evaluated the relationship between the intake of quercetin and the incidence rate of the ovarian cancer among the nursing staff. The data collected from these results revealed that quercetin intake had a nonsignificant 29% decrease in ovarian cancer risk compared to women with the lowest intake of quercetin.

## 9. Conclusion and Future Perspectives

The studies presented here suggest the potential effects of quercetin in cancer therapy. Numerous in vitro and in vivo experiments have shown various mechanisms of action that could suppress multiple oncogenic signaling pathways. Quercetin is safe with no reported toxicity when applied for the treatment of human cancer. Since quercetin and its derivatives have great benefits, it is the need of the hour to investigate further the effects of these molecules in the prevention and intervention of cancer. However, there is still no conclusive evidence regarding its exact mode of action in order to enhance its clinical application in the treatment of human cancer. Therefore, the future perspective of research should concentrate on the evaluation of quercetin’s precise mechanisms of action. Similarly, it is necessary to perform more clinical studies on the efficacy and bioavailability of quercetin in biological systems for the future use in human population, especially in the treatment of cancer. Moreover, the conversions of quercetin to its metabolites must be considered while assessing the efficacy and bioavailability of quercetin for further pharmacological use. The conjugation of xenobiotics with quercetin alters the reactivity of quercetin, but there are some metabolites of quercetin in conjugated form which showed beneficial biological activities. It is important to investigate further mechanisms of action of quercetin, especially in terms of suppressing carcinogenicity in rodents. The results obtained from the current epidemiological studies shows that there is shortage of evidence of quercetin intake for the prevention of human cancer. There is a need to conduct further epidemiological studies to evaluate the role of quercetin in human cancer prevention. Updating the database related to dietary flavonoids will deliver significant information for future epidemiological studies. The use of quercetin in the field of pharmaceuticals is limited because of its poor water solubility and oral bioavailability. In order to enhance the solubility and bioavailability of quercetin inside human body, various scientific approaches have been taken into consideration, including the application of novel drug delivery systems such as nanoparticles and liposomes. These and additional approaches may help us to understand the full potential of quercetin in cancer prevention and therapy.

## Figures and Tables

**Table 1:** In vitro anticancer effects of quercetin and its analogs.

Compound tested	Cell lines	Effects	Mechanisms	References
Quercetin	MCF-7, HCC1937, SK-Br3, 4T1, MDA-MB-231	Induced apoptosis	↑Bcl-2,↓Bax expression,↓Her-2, inhibition of PI3K-Akt pathway	[71]
Quercetin	MIA PaCa-2, BxPC-3	Inhibited proliferation	↓Her-2, regulation of Wnt/β-catenin	[72]
Quercetin	CX-1, SW480, HT-29, HCT116	Inhibited proliferation	↓HIF-1κ, regulation of Wnt/β-catenin	[73]
Quercetin	LNCaP, PC-3	Inhibited proliferation	↓VEGF secretion, ↓mRNA levels	[74]
Quercetin	HepG2	Inhibited proliferation	↓PI3K,↓PKC	[75]
Rutin	ACC	Inhibited proliferation	↓PI3K,↓Akt,↓IKK-α,↓NF-κB	[76]
Rutin	SKOV3	Inhibited cell growth	↓Cyclin D1	[77]
Rutin	HeLa	Inhibited cell growth	↑p53,↓NF-κB	[78]
Quercetin	A549	Inhibited cell growth	↓cdk1,↓cyclin B	[79]
Quercetin	JB6 P+	Inhibited cell migration	Regulation of p13K/Akt	[5]
Quercetin	U373MG	Inhibited cell migration	↑caspase-7,↑JNK,↑p53	[80]

**Table 2:** In vivo anticancer effects of quercetin and its analogs.

Compound tested	Animal models	Effects	Mechanisms	Dose	Duration	References
Quercetin	FemaleCF1 mice	Retarded tumor growth	↓PCNA;↑mmu-miR-205-5P	8 g/kg/day (diet)	42 days	[88]
Quercetin	Male F344 rats	Inhibited tumor growth	↓EphA2;↓PI3K;↓MMP-2;↓MMP-9	100 mg/kg (i.p.)	18 days	[89]
Rutin	Male F344 rats	Suppressed tumor growth	↓ACF	25 mg/kg (i.p.)	28 days	[89]
Quercetin	Female CD-1 mice	Inhibited tumor nodule formation	↓papilloma	3–6 mg/kg (p.o.)	14 days	[90]
Quercetin	Male Swiss mice	Inhibited tumor nodule formation	↓AD	6 mg/kg (i.p.)	2 times/week 21 days	[91]
Quercetin	Female Sprague-Dawley rats	Reduced tumor volume	↓ADC	17.5 mg/kg (i.v.)	2 times/week for 24 days	[92]

i.p., intraperitoneal; i.v., intravenous; p.o., per os.
